# A veil of ignorance can promote fairness in a mammal society

**DOI:** 10.1038/s41467-021-23910-6

**Published:** 2021-06-23

**Authors:** H. H. Marshall, R. A. Johnstone, F. J. Thompson, H. J. Nichols, D. Wells, J. I. Hoffman, G. Kalema-Zikusoka, J. L. Sanderson, E. I. K. Vitikainen, J. D. Blount, M. A. Cant

**Affiliations:** 1 Centre for Ecology and Conservation, University of Exeter, Penryn, UK; 2 Centre for Research in Ecology, Evolution and Behaviour, University of Roehampton, London, UK; 3 Department of Zoology, University of Cambridge, Cambridge, UK; 4 Swansea University, Swansea, UK; 5 Department of Animal Behaviour, Bielefeld University, Bielefeld, Germany; 6 School of Natural Science and Psychology, Liverpool John Moores University, Liverpool, UK; 7 British Antarctic Survey, Cambridge, UK; 8 Conservation Through Public Health, Entebbe, Uganda; 9 Faculty of Biological and Environmental Sciences, University of Helsinki, Helsinki, Finland; 10 Institute for Advanced Study, Berlin, Germany

**Keywords:** Evolutionary theory, Social evolution

## Abstract

Rawls argued that fairness in human societies can be achieved if decisions about the distribution of societal rewards are made from behind a veil of ignorance, which obscures the personal gains that result. Whether ignorance promotes fairness in animal societies, that is, the distribution of resources to reduce inequality, is unknown. Here we show experimentally that cooperatively breeding banded mongooses, acting from behind a veil of ignorance over kinship, allocate postnatal care in a way that reduces inequality among offspring, in the manner predicted by a Rawlsian model of cooperation. In this society synchronized reproduction leaves adults in a group ignorant of the individual parentage of their communal young. We provisioned half of the mothers in each mongoose group during pregnancy, leaving the other half as matched controls, thus increasing inequality among mothers and increasing the amount of variation in offspring birth weight in communal litters. After birth, fed mothers provided extra care to the offspring of unfed mothers, not their own young, which levelled up initial size inequalities among the offspring and equalized their survival to adulthood. Our findings suggest that a classic idea of moral philosophy also applies to the evolution of cooperation in biological systems.

## Abstract (web summary)

Obscuring knowledge of personal gains from individuals can theoretically maintain fairness in a cooperative group. Experiments show that wild, cooperatively breeding banded mongooses uncertain of kinship allocate postnatal care in a way that reduces inequality among offspring, suggesting a classic idea of moral philosophy can apply in biological systems.

## Introduction

The idea that impartiality or ignorance on the part of decision-makers promotes cooperation and fairness in human societies has a long pedigree in philosophy and economics^1–3^. Individuals that are blind to their own gains are predicted to allocate resources for the good of the group rather than themselves^1^, typically reducing inequality^2^. In biology, an analogous argument has been proposed as a mechanism for the evolution of cooperation among self-interested agents^4,5^. Meiosis, for example, ensures that each allele has an equal chance of ending up in any given offspring, creating a “Mendelian veil of ignorance”^4^, which aligns the fitness interests of each gene with that of the organism^4,6–8^. In insect societies, uncertainty over relatedness promotes cooperative behaviour: workers cooperate to raise the offspring of other workers when relatedness to offspring is uncertain, but kill such offspring when they can discriminate worker-laid vs. queen-laid eggs^5^. Our aim was to understand if a veil of ignorance over kinship can also promote fairness in an animal society, in the sense of a redistribution of resources to reduce initial inequalities, analogous to Rawls’^2^ redistributive concept of fairness in human societies.

We studied fairness in the allocation of postnatal care in wild groups of cooperatively breeding banded mongooses *Mungos mungo*^9^. The veil of ignorance arises in this system because multiple females (mean ± s.d. = 5.0 ± 2.6, *n* = 84 litters) synchronize birth to the same morning in a shared underground den. There is good evidence that this extreme birth synchrony removes cues to the parentage of offspring. First, mothers are observed to suckle pups in synchronously produced litters without any apparent discrimination, and pups move from female to female to suckle in a single suckling session^10^. Second, after pups emerge from the den parents and other helpers do not preferentially care for more closely related offspring in communal litters^11^. Third, on rare occasions when cues to parentage are available (e.g., in the minority of breeding attempts that are asynchronous^12^, or when older females are reproductively suppressed using contraceptives^13^), females kill the pups of other females rather than care for them, suggesting that such cues are absent in natural, synchronous litters.

The communal litter (hereafter “litter”) is suckled underground and guarded at the den by babysitters for the first month, after which the pups emerge from the den and form one-to-one caring relationships with particular adults, called escorts^11,14,15^. Escorts, who can be any adult male or female in the group, feed and protect the pup in their care until it reaches nutritional independence at around 90 days old (see “Methods”). Both escorts and pups contribute to maintaining the association^14,16–18^. Escorts individually recognize and preferentially respond to the calls of “their” particular pup^16,18^, and actively seek out their pup if it becomes separated or lost^14^. Escorts almost exclusively provision the pup with which they are associated^14,19^, and young pups receive almost all their food from their escort^19^. Pups, for their part, aggressively defend access to their escort^14,15,17^ and beg continuously for food^16^. Pups who receive more escorting are heavier at independence^20^ and heavier pups are more likely to survive^21^.

Below we develop a simple game theoretical model to investigate how the veil of ignorance over parentage in banded mongoose communal litters might influence fairness in the distribution of postnatal care among the offspring. We test our model by creating inequalities among helpers and among offspring through the targeted provisioning of pregnant females, and then measuring whether mothers and helpers act to reduce or amplify these inequalities.

## Results and discussion

**Fig. 1:**
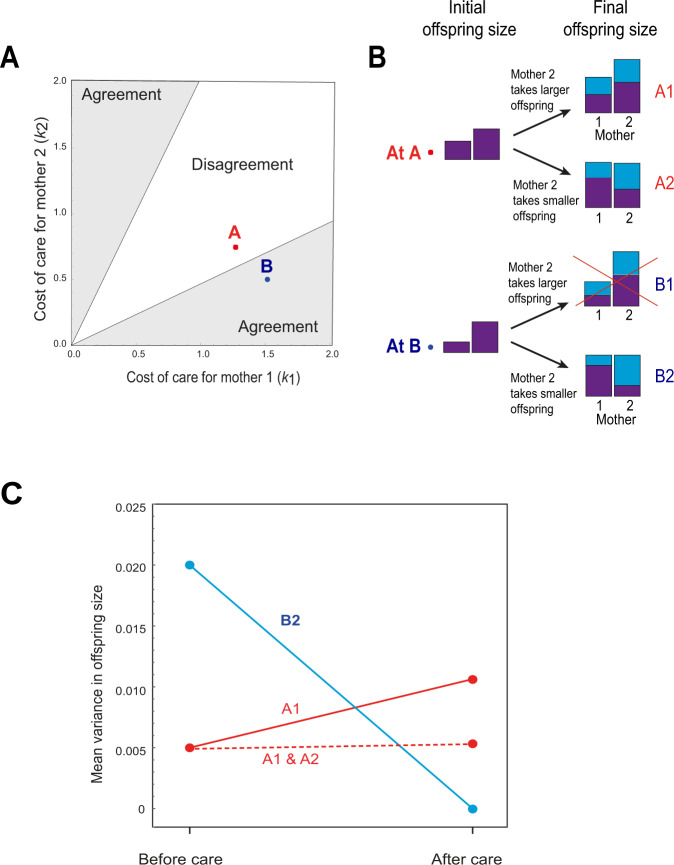
Model of parental investment from behind a veil of ignorance. **A** Zones of agreement and disagreement over which mother cares for which offspring (see main text), when *b*(*x*) = *x*(1 − *x*/2) (for *x* ≤ 1) and *c*(*k;y*) = *k y*^2^. Two illustrative sets of parameter values are marked: point A (in the zone of disagreement) where we assume that initial asymmetries between parents and between offspring are smaller (*k*_1_ = 1.25, *k*_2_ = 0.75, *x*_hi_ = 0.25, *x*_lo_ = 0.15) and point B (in the zone of agreement) where initial asymmetries between parents and between offspring are larger (*k*_1_ = 1.5, *k*_2_ = 0.5, *x*_hi_ = 0.3, *x*_lo_ = 0.1). **B** The predicted prenatal (purple shading) and postnatal (light blue shading) investment received by each offspring given each of the two sets of parameter values A and B. For each set of parameter values A and B we show the outcomes when (i) the inferior carer, here mother 1, looks after the initially smaller offspring (outcomes A1 and B1), and when (ii) mother 1 looks after the initially larger offspring (outcomes A2 and B2). At point A, the mothers disagree over who should care for whom, so neither outcome is universally preferred; at point B, outcome B2 is preferred by both parents, so outcome B1 is never chosen and is crossed out. **C** Variance in offspring size before and after postnatal investment at point A, assuming either outcome A1 (solid red line) or that outcomes A1 and A2 are equally probable (dashed red line), and at point B, assuming the mutually preferred outcome B2 (solid blue line).

We constructed and analyzed a simple model of investment in offspring care (Fig. 1). This model is highly simplified compared to our empirical system, but is useful to illustrate how the veil affects care decisions and to derive testable predictions. Consider two unrelated mothers, who may differ in quality or condition, have each produced a single offspring. Note, we use the example of two mothers for simplicity, but our model could be applied to carers of either sex, and to parental and alloparental helpers. Similarly, we assume unrelated mothers for illustrative purposes, but in the Supplementary Information (SI) we consider a model in which mothers are related. Each mother must now take on the care of one or other of the two young, which may differ in initial size (denoted *x*_*i*_ for offspring *i*). A mother may choose to invest a variable amount of effort (*y*_*i*_ for mother *i*) in caring for the offspring she accepts. The survival of offspring *i*, when raised by mother *j*, is a smoothly increasing but decelerating function *b*(*x*_*i*_ + *y*_*j*_) of the total investment it receives pre- and post-natally, while the mother incurs a cost to her future reproductive success *c*(*k*_*j*_; *y*_*j*_) that is a smoothly increasing and accelerating function of *y*_*j*_. The parameter *k*_*j*_ determines how steeply the cost of care increases with investment for mother *j*.

In this model, if parentage of the young were known to the mothers (i.e., the “no veil” case), each would choose to care for its own offspring, regardless of any differences in offspring size or parental condition (i.e., selection favors investment strictly according to parentage).

Suppose, however, that a veil of ignorance obscures the relationships between parents and young. Where mothers are in similar condition, and hence pay similar costs of care, there arises a conflict of interest over who cares for whom, as each mother stands to gain if she raises the larger of the two offspring herself (the unshaded “zone of disagreement” shown in Fig. 1A; see SI for details). If the costs of care are substantially lower for one mother, because she is in significantly better condition, both prefer the outcome in which this superior mother raises the smaller offspring, regardless of parentage, resulting in “care according to need” (the shaded “zones of agreement” shown in Fig. 1A). This arrangement increases the expected fitness of both carers, so that they both agree on who should care for whom (see SI). While relatedness between mothers can also, in theory, lead to exchange of offspring, in the absence of a veil such exchange is mutually agreeable only for implausibly high levels of relatedness and for implausibly large asymmetries in initial size between young. For example, given a three-fold difference in size between offspring, exchange requires relatedness values of ~0.7 or greater, and this relatedness requirement is even higher where offspring are more similar in size. In the banded mongoose system, the size ratio of largest to smallest offspring equals 1.44 ± 0.29 (mean ± s.d.), and median genetic relatedness between females equals 0.24 (IQR = 0.05–0.37; see “Methods” and SI for details).

What are the predicted consequences of the veil of ignorance for inequalities among offspring? When asymmetries in initial offspring size and in parental condition are small, so that the system falls within the zone of disagreement (e.g., point A in Fig. 1A), the effect of care on inequality among offspring (i.e., final offspring size or total investment) depends on how conflict over the choice of offspring is resolved. If mothers in better condition succeed in obtaining their preferred outcome (outcome A1 in Fig. 1B) the small parental asymmetry weakly reinforces the initial inequality among offspring (the solid red line in Fig. 1C); alternatively if either the superior or the inferior mother is equally likely to obtain her preferred outcome (so that outcomes A1 and A2 in Fig. 1B are equally probable) there is on average little change in inequality over the care period (dashed red line in Fig. 1C). By contrast, when asymmetries in initial offspring size and in parental condition are large, so that the system falls within the zone of agreement (e.g., point B in Fig. 1A), the superior mother always (by agreement) cares for the smaller, needier offspring, with the result that the large parental asymmetry should level out the initial inequality among offspring (outcome B2 in Fig. 1B; solid blue line in Fig. 1C). Paradoxically, therefore, we predict that behind the veil of ignorance, greater inequalities between parents lead to a fairer outcome for offspring.

We created early-life asymmetries among mongoose carers and their offspring by provisioning half the pregnant females in each of 7 groups daily with 50g of cooked egg, while leaving the other half of pregnant females as within-group controls (see “Methods” and Fig. S5 in SI). We did this for 34 communal breeding attempts involving 101 fed and 97 unfed mothers. In between experimentally manipulated breeding attempts we left each group unmanipulated for one full breeding attempt. We followed changes in maternal and offspring weight and patterns of postnatal care across three phases of development: pregnancy (5–30 days before birth), post-pregnancy (5–19 days after birth), and escorting (30–90 days after birth); we also measured offspring survival to adulthood. Following the predictions of our model, we asked four questions: (1) Are mothers that were provisioned during pregnancy in better condition in the postnatal period compared to control mothers? (2) Do fed females produce larger offspring compared to control mothers? (3) Do mothers then allocate postnatal care according to parentage (investing in their own young regardless of differences in initial offspring size or parental condition), or according to need (those mothers in better condition investing in smaller offspring regardless of parentage), and does increased care translate into more resources for offspring? (4) Do these patterns of maternal allocation amplify or reduce inequality among offspring?

**Fig. 2:**
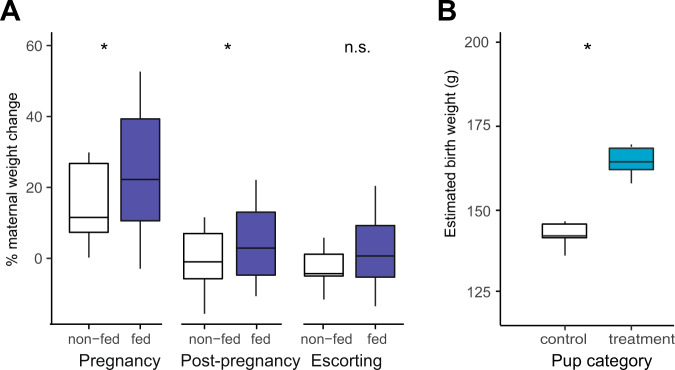
Effect of gestational feeding on weight of mothers and offspring. **A** Percent weight change (relative to conception weight) of non-fed (no shading) and fed (dark blue) mothers during pregnancy (5–30 days before birth), in the post-pregnancy period (5–19 days after birth) and escorting period (30–90 days after birth). **B** Birth weight predicted by our model of pup growth for “control” offspring produced by non-fed mothers (no shading) and “treatment” offspring produced by fed mothers (light blue) offspring. Filled boxes show the interquartile range (IQR) and median (internal horizontal line). Whiskers (vertical lines) show the extent of observations outside the IQR to a maximum of 1.5x IQR. Other legends: n.s. *p* > 0.05; *p < 0.05.

First, on average mothers that were provisioned prenatally (“fed”) gained more weight during their pregnancy than control (“non-fed”) mothers in the same breeding attempt (24.1 ± 4.5% vs. 15.1 ± 3.7% weight change, *n* = 45; post-hoc Tukey’s test (PHT): *z* = 2.42, *p* = 0.039), and remained heavier in the post-pregnancy period (4.36 ± 2.78% vs. −0.19 ± 3.36% weight change, *n* = 41; PHT: *z* = 2.68, *p* = 0.019; Fig. 2A). By the end of the escorting period, however, prenatally provisioned mothers were no heavier than control mothers (3.33 ± 3.11% vs. −1.95 ± 2.08% weight change, n = 43; PHT: *z* = 1.22, *p* = 0.44; Fig. 2A), suggesting that the extra resources assimilated by fed mothers during pregnancy had been used up by the end of the postnatal care period.

Second, the pups of fed mothers (“treatment pups”) were estimated by our model of pup growth (Table S5) to be heavier at birth than the “control” pups of non-fed mothers (treatment = 164.9 ± 3.5 g, control = 142.0 ± 3.2 g; *z* = 2.13, *p* = 0.03, *n* = 293; Fig. 2B). Provisioning of half the breeding mothers in each group during pregnancy resulted in greater within-litter variance and inequality in pup weight at the start of the escorting period compared to unmanipulated litters in which no provisioning occurred (Mann–Whitney test, *U* = 16, *p* = 0.029, *n* = 12). Thus, our experiment had the effect of increasing inequality in body weight among the members of the communal litter, prior to the start of the escorting period.

**Fig. 3:**
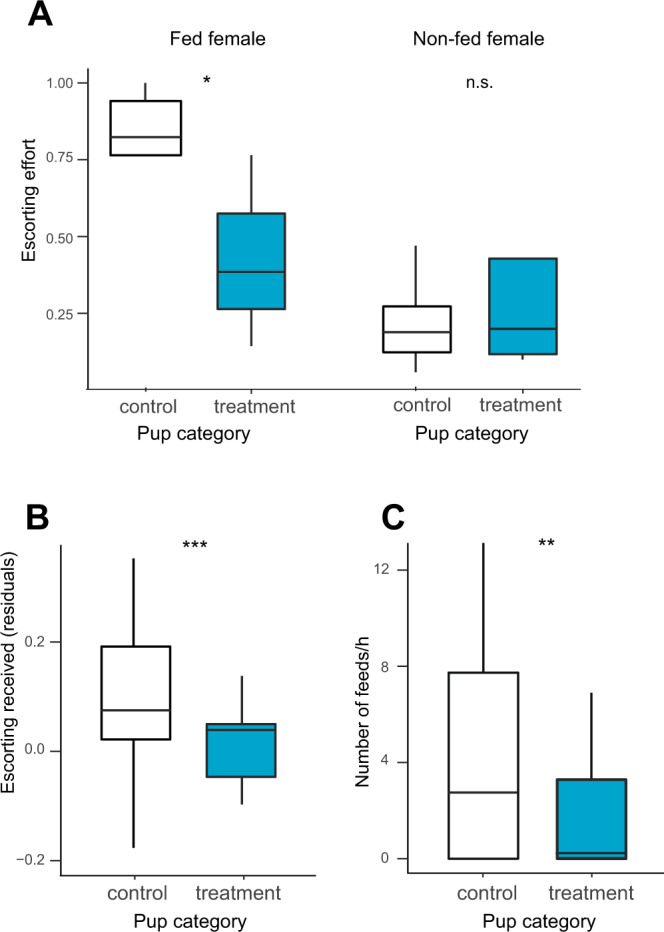
Pattern of postnatal care. **A** Escorting effort (proportion of time spent escorting) directed to control (unshaded) and treatment (sky blue) pups by fed females and non-fed females. **B** Overall escorting received by control and treatment pups (residual values plotted once other variables are controlled for in our statistical model, Table S6). **C** Rates at which the control and treatment pups were fed by their escort. Boxes show the interquartile range (IQR) and median (internal horizontal line). Whiskers (vertical lines) show the extent of observations outside the IQR to a maximum of 1.5x IQR. Other legends: n.s.: *p* > 0.05; *p < 0.05; **p < 0.01; ***p < 0.001.

Third, fed mothers contributed more on average to escorting than non-fed mothers (PHT: *β* ± s.e. = 1.44 ± 0.42, *z* = 3.39, *p* = 0.002, *n* = 34), or mothers in unmanipulated breeding attempts (PHT: *β* ± s.e. = 1.91 ± 0.37, *z* = 5.11, *p* = 6.33 × 10^−7^, *n* = 34). Moreover, fed mothers provided elevated escorting effort to the offspring of non-fed mothers (i.e., control pups), rather than their own genetic offspring (PHT: *β* ± s.e. = 2.52 ± 1.08, *z* = 2.32, *p* = 0.02, *n* = 21; Fig. 3A). Non-fed mothers, by contrast, showed no discrimination between control and treatment pups (PHT: *β* ± s.e. = 0.12 ± 0.77, *z* = 0.16, *p* = 0.88, *n* = 21; Fig. 3A). In other words, fed mothers invested in needier offspring to whom they were unrelated, rather than in their own young. Adult males also increased their escorting effort in experimental breeding attempts compared to unmanipulated breeding attempts (PHT: *β* ± s.e. = 0.97 ± 0.35, *z* = 2.76, *p* = 0.006, *n* = 110). Like non-fed mothers, however, male care patterns were not different for control vs. treatment pups (PHT: *β* ± s.e. = 1.31 ± 0.79, *z* = 1.65, *p* = 0.10, *n* = 56). Thus, fed mothers allocated postnatal care according to need (perhaps based on cues such as pup size or begging rate) in a way that males and non-fed females did not.

**Fig. 4:**
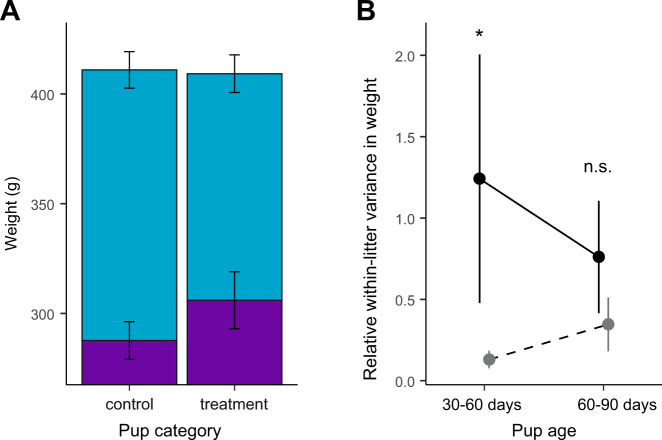
Change in inequality among pups due to postnatal care. **A** Initial weight (purple bars) and weight at independence (light blue stacked bars) of control and treatment pups. **B** Relative within-litter variance in pup weight in experimental litters (black, solid line) and in unmanipulated litters (gray, dashed line) in which no females were fed during pregnancy. All values and error bars are the mean ± standard error. n.s.: *p* > 0.05; *p < 0.05.

Fourth, as a result of receiving extra investment from fed mothers, control pups received more escorting overall than treatment pups (PHT: *β* ± s.e. = 0.81 ± 0.23, *z* = 3.59, *p* = 9.13 × 10^−4^, *n* = 71; Fig. 3B) and were fed by their escort at a greater rate (*β* ± s.e. = 0.85 ± 0.28, *z* = 3.09, *p* = 0.005, *n* = 79, Fig. 3C). Consequently, they grew more rapidly over the escorting period than treatment pups (or, indeed, unmanipulated pups) so that, by the end of the escorting period (age 60–90 days), the initial body mass disparity between control and treatment pups had been eradicated (pup body weight at 30 days: treatment = 315.5 ± 11.6 g, control = 299.4 ± 7.9 g; age 60–90 days: treatment = 413.6 ± 8.9 g, control = 420.8 ± 8.6 g, treatment x age PHT: *z* = 2.68, *p* = 0.020, *n* = 293; Fig. 4A). Inequality in pup body weight, which had been significantly elevated by our experimental manipulation (inequality in experimental litters at age 30–60 days vs. unmanipulated litters: *U* = 16, *p* = 0.029, *n* = 12), was levelled by the end of the escorting period (age 60–90 days: *U* = 13, *p* = 0.38, *n* = 12; Fig. 4B). In terms of survival, the supernormal levels of postnatal care received by control pups (which also exceeded the care received by pups in unmanipulated litters, SI) appeared to compensate fully for their birth weight disadvantage, since there was no difference between control and treatment pups in survival to 90 days, 6 months or 1 year (90 days: *χ*^2^ = 1.07, *p* = 0.59; 6 months: *χ*^2^ = 2.21, *p* = 0.33; 1 year: *χ*^2^ = 2.51, *p* = 0.29; *n* = 128, Table S7).

We have shown using an experimental approach that, when a subset of mothers are given additional resources, they invest disproportionately in smaller, control offspring, not their own young. This pattern of investment is the opposite to that expected if mothers invest according to parentage. However, as our model demonstrates, this is the predicted pattern of investment from behind a veil of ignorance over kinship. Parents in good condition are selected to invest according to need, and to provide the disproportionate investment required to compensate for an initial disadvantage. The model also helps reconcile our experimental results with our long-term data showing that, in natural breeding attempts (in which there is less variation in maternal condition and offspring size), it is larger rather than smaller offspring that receive more escorting^11,22^. Where asymmetries between mothers in condition are less extreme, our model predicts that mothers will prefer to invest in larger, rather than smaller young, amplifying the inequality in the communal litter (Fig. 1). Our findings are as expected if our experiment shifted asymmetries in maternal condition from the zone of disagreement into the zone of agreement in Fig. 1A, in which all parties agree that better condition mothers should care for poorer condition offspring.

It is important to ask whether our results could be explained by kin selection, given that co-breeding females are genetically related to each other’s offspring (specifically, median relatedness among adult females is 0.24, IQR = 0.05–0.37). For example, fed mothers might in theory favor investing in smaller offspring to whom they are related, if these smaller offspring stand to benefit relatively more from additional care than their own larger young. In the Supplementary Information we explored this alternative hypothesis by adapting our model to the case where co-breeding females are genetic relatives. Our model shows that in the absence of a veil of ignorance, helping according to need rather than kinship can indeed evolve, but only at implausibly high levels of relatedness among breeders (*r* > 0.7 for reasonable assumptions about the costs and benefits of care). Moreover, our data show that median relatedness between female escorts and their pups in experimental litters was 0.11 (IQR = −0.04–0.30). Thus, by caring for control pups, fed mothers targeted their care at offspring that were far less closely related to themselves than their own young, which cannot plausibly be explained as the result of kin selection. In contrast this pattern is readily explained by our model based on the veil of ignorance.

Other studies of human and non-human animals have investigated fairness preferences by measuring aversion to different forms of inequity in choice tasks. In these studies human and non-human subjects commonly reject rewards or show negative behavioral responses when presented with inequality that is disadvantageous to themselves^23–26^. However, only humans typically pay costs to redress inequalities that are advantageous to themselves^26,27^. In young children, for example, focal subjects sacrifice rewards to achieve equality with a social partner, whereas chimpanzees presented with an equivalent task do not^27^. It has been argued that this human propensity for redistributive fairness is a consequence of our evolutionary history of mutual cooperation^27,28^. In repeated interactions in which there is uncertainty about payoffs or roles in social interactions, egalitarian sharing of resources insures each actor against the risk of finding itself in a disadvantaged position^3,28–30^. Fairness in our model of parental investment arises for the same reason: from behind a veil of ignorance over kinship, mothers invest postnatally to minimize the risk that their own offspring will face a disadvantage. Our findings show that uncertainty about kinship in a non-human species can lead to a redistribution of resources to reduce inequality, consistent with the proposed role of the veil of ignorance in the evolution of human fairness norms^31^.

## Methods

### Study system

Our study was carried out on a population of wild banded mongooses (*Mungos mungo*) on the Mweya Peninsula, Queen Elizabeth National Park, Uganda (0°12′S, 29°54′E). As part of a long-term research project, individual-based life history data have been collected on this population since 1995.

Banded mongooses are diurnal carnivores (<2 kg) that live in stable, mixed-sex groups of around 10 to 30 individuals. Individuals sexually mature around the age of one year old^32^ and all sexually mature individuals within a group have the opportunity to reproduce to some extent^33,34^. Their average lifespan is around 3.5 years (males = 42 months, females = 38 months, max = 149 months)^32^. At our equatorial study site reproduction occurs all year round and is not synchronized between groups. Reproduction is, however, highly synchronized within groups: around four times per year, all pregnant females in a group give birth in an underground den to a large communal litter, usually on the same morning^12,35^. Pups remain in the den for approximately their first 30 days, after which they move with the rest of the group and are cared for by adult “escorts” who feed, carry, groom and protect the pup from predators for around a further 60 days^15,36^. We are able to individually recognize the mongooses in our study population using unique hair-shave patterns or colored collars. Radio collars weighing 26 to 30 g (Sirtrack Ltd, Havelock North, New Zealand) with a 20 cm whip antenna (Biotrack Ltd, Dorset, UK) are fitted to one to two individuals in each group to allow them to be located.

### Data collection

We visited our study groups for at least 20 min every 1–3 days, during which we noted the presence or absence of individuals. We could distinguish between absences due to dispersal from the group and due to mortality since in banded mongooses dispersal involves the simultaneous eviction of multiple individuals from the group, often with a conspicuous period of aggression within the group beforehand^37,38^. By contrast, mortality events are observed directly or inferred from the permanent disappearance of single individuals. We were able to weigh most individuals once a week in the morning before foraging started by training them to step on electronic scales in return for a small milk reward. We identified female pregnancy by visual swelling of the abdomen and confirmed this through palpation and ultrasound scans^39^. Births occur overnight in an underground den, and were identified by the absence of pregnant females the following morning and a subsequent change in their body shape and mass loss^12,35^. To assign parentage, DNA was extracted from 2 mm skin samples taken from individuals when they were first trapped (either as newly emerged pups or newly arrived immigrants). This DNA was then genotyped using a panel of 43 polymorphic microsatellite markers (see further details of DNA analysis, parentage assignment and trapping procedure in refs. ^40,41^).

Pups emerge from the underground den at 30 days of age and are cooperatively cared for by adult “escorts” who feed, carry, groom and protect the pup from predators for around a further 60 days, hereafter termed the “escorting period”^15,36^. These escorting relationships involve a single pup being cared for by a single adult carer, though pups may switch between escorts across the escorting period^42^. During the escorting period groups were visited every day for at least 20 min. We measured the amount of escorting received by a pup as the proportion of group visits they were observed being escorted by an adult. Pups were defined as being escorted if they were within 30 cm of the same adult for more than 50% of the period of observation^15,43^. In addition, we also conducted 20 min focal follows^44^ on pups during the escorting period, continuously recording when they were fed by their adult escort and, at 60 s intervals, the identity of their nearest adult neighbor. Pups were weighed at age 30–60 days (median age at weighing = 49 days, IQR = 46–56, *n* = 83 weights) and again at 60–90 days old (median age at second weighing = 77 days, IQR = 67–82, *n* = 210).

### Experimental design: inducing asymmetries among adults and pups

We induced early-life asymmetries among banded mongoose pups by manipulating the food resources available to pregnant females. As outlined above, female mongoose reproduction within mongoose groups is highly synchronized, with all females usually giving birth on the same day. Our experimental design (Fig. S5) took advantage of this synchronous reproduction to create a split-plot design where half of the pregnant females in a given breeding attempt were assigned as provisioned (“fed”) and the other half were assigned as controls (“non-fed”). Females were assigned to each experimental category in age-matched pairs after pregnancy was confirmed by ultrasound and palpation at around three weeks after conception (see trapping details above). If neither female in a pair had been involved in a previously manipulated breeding attempt, as either a fed or non-fed female, then they were randomly assigned as either fed or non-fed. If they had been part of a previously manipulated breeding attempt they were assigned to the opposite category to that assigned previously. If both females had been in the same category previously then they were re-paired with the next-nearest aged females. In breeding attempts with an odd number of pregnant females, the unpaired female was randomly assigned a category if she had not been part of a manipulated breeding attempt before, or, if she had, she was assigned to the opposite category to that assigned previously. Across all groups, each manipulated breeding attempt was followed by an “unmanipulated” breeding attempt where no females were provisioned as a further control and to ensure the effects of the provisioning fully dissipated before the next manipulation (Fig. S5).

During manipulated breeding attempts fed females were provisioned with an average of 50 g of egg per day until birth (*n* = 101 fed female pregnancies), whilst non-fed females were approached but not provisioned (*n* = 97 non-fed female pregnancies). Provisioning began around 2–4 days after pregnancy had been confirmed using ultrasound and continued up to the day before parturition (mean ± s.d. provisioning time = 24 ± 9 days). To ensure fed females were not able to predict the amount and timing of provisioning (which might influence their natural foraging behavior) we randomized the amount of egg a fed female received each day (0, 50, or 100 grams) and the time of day she received this egg (a.m. or p.m.).

In total, we manipulated 34 breeding attempts in 7 banded mongoose groups across a three-year period (spanning 2013–2016). The resulting dataset corresponded to a total of 101 fed female pregnancies and 97 non-fed female pregnancies. Pups emerge from the underground den at around 30 days after birth and our manipulated breeding attempts produced 50 pups from fed females (hereafter “treatment pups”) and 50 from non-fed females (“control pups”; see details of parentage assignment above).

Median relatedness between adults in these breeding attempts was 0.22 (IQR = 0.07–0.36; manipulated: 0.23, IQR = 0.08–0.37; unmanipulated: 0.21, IQR = 0.06–0.35; *n* = 8191 dyads across 49 breeding attempts). Adult females had a median relatedness of 0.24 (IQR = 0.05–0.37; manipulated: 0.24, IQR = 0.08–0.38; unmanipulated: 0.23, IQR = 0.04–0.37 *n* = 1134 dyads across 49 breeding attempts).

### Statistical methods

We tested the effect of our experimental prenatal provisioning on (i) female condition and (ii) how they allocated their postnatal care (escorting) using general linear mixed-effects models. First, to test the effect of supplementary provisioning on female condition we calculated each female’s percentage change in weight over the course of the breeding attempt. Banded mongoose gestation lasts for 59–62 days^10^ and so we used a female’s weight at 67–74 days before birth as their pre-pregnancy baseline weight. For each female we measured their percentage change in weight from this baseline during:

- Pregnancy, using the latest weight record 5–30 days before birth (mean ± s.d. = 10.8 ± 4.25 days before birth, *n* = 45)
- Post-pregnancy, using the latest weight record 5–19 days after birth (mean ± s.d. = 15.6 ± 2.22 days after birth, *n* = 41)
- Escorting period, using the latest weight record 30–90 days after birth (mean ± s.d. = 77.6 ± 14.9 days after birth, *n* = 43)

For each of these periods we tested the effect of our experimental provisioning using a linear mixed-effects model with a normal error structure. This included experimental category (fed, non-fed or unmanipulated) as a fixed effect and individual, breeding attempt and social group ID as random intercepts. In all cases the model residuals were normally distributed with constant variance (see Table S1 for sample sizes).

Second, to test the effect of experimental prenatal provisioning on adult escorting behavior we fitted models predicting, separately, female and male total escorting effort across the escorting period. We measured escorting effort as the proportion of all group visits that an individual was observed escorting. These models included experimental category (female model: fed, non-fed or unmanipulated; male model: manipulated or unmanipulated breeding attempt), the ratio of adults (potential escorts) to pups and individual age as fixed effects, and individual, breeding attempt and social group ID as random intercepts (see Table S1 for sample sizes). We then tested how escorting was allocated among pups by constructing models predicting the amount of escorting effort that fed and non-fed females, and males, directed toward each pup in the manipulated litters. Our model of female escorting allocation included female experimental category (fed or non-fed) and pup experimental category (treatment or control), as well as the interaction between these two variables as fixed effects. Our model of male escorting allocation included pup experimental category (treatment or control) as a fixed effect. Both the female and male escorting allocation models also included pup sex, the ratio of adults (potential escorts) to pups and adult age as fixed effects, and pup, adult, breeding attempt and social group ID as random intercepts. We used a binomial error structure and logit link function in all of the escorting effort and allocation models (see Table S1 for sample sizes).

We then tested the effect of our experimental prenatal provisioning on pups’ (i) weight and growth, (ii) the amount of escorting they received, and (iii) their survival. First, to test the effect of maternal provisioning on pup weight and growth we constructed a model predicting pup weight at 30–90 days old. This model included pup experimental category (treatment, control or unmanipulated), pup sex, pup age (days) and the ratio of adults (potential escorts) to pups as fixed effects. It also included the interaction between experimental group and age to test for differing growth rates in different experimental categories. Pup, breeding attempt, and social group ID were included as random intercepts. Initial data and model exploration revealed overdispersion so we also included an observation-level random effect in the model and fitted it using a Poisson-lognormal error structure^45^ (see Table S1 for sample sizes). As pups are kept underground for the first few weeks of life, we also used this model to estimate birth weight of pups (i.e., at day zero). In addition, to evaluate how the experimental provisioning influenced inequality amongst pups across the escorting period we compared the relative within-litter variance in pup weight of manipulated and unmanipulated breeding attempts when pups were 30–60 days (*n* = 8 breeding attempts) and 60–90 days old (*n* = 9). Relative within-litter variance in weight was calculated as the absolute within-litter variance in weight divided by the total variance in weight across all litters. We used Mann–Whitney tests to compare the relative within-litter variances (this excluded breeding attempts containing fewer than four pups).

Second, we fitted a model testing the effect of maternal provisioning on the amount of escorting received by pups. The total amount of escorting received by a pup was measured as the proportion of all group visits in which that pup was observed being escorted. We included pup experimental category (treatment, control or unmanipulated), pup sex and the ratio of adults (potential escorts) to pups as fixed effects, and breeding attempt and social group ID as random intercepts. The model was constructed using a binomial error structure and a logit link function (see Table S1 for sample sizes). We also constructed a model of the rate at which escorts were observed feeding pups during each focal follow (number of feeds/hour). This model included pup experimental category (treatment, control or unmanipulated) and the strength of the relationship between pup and escort as fixed effects. Relationship strength was measured as the proportion of 60 s scans the escort appeared in. We included pup, breeding attempt and social group ID as random intercepts. Initial data and model exploration revealed overdispersion so we also included an observation-level random effect in the model and fitted the model using a Poisson-lognormal error structure^45^ (see Table S1 for sample sizes).

Third, we constructed models testing the effect of maternal provisioning on pup survival to 90 days (end of the escorting period), 180 days (sub-adulthood) and 365 days old (adulthood). We used Cox proportional hazards mixed-effects models predicting pup survival using left-truncated and right-censored datasets for each time period. These included experimental category (treatment, control, or unmanipulated), pup sex and the ratio of adults (potential escorts) to pups as fixed effects, and breeding attempt and social group ID as random effects. The models’ residuals were visually checked for non-proportional hazards, influential observations, and nonlinearities (see Table S1 for sample sizes).

For all of our generalized linear and Cox proportional hazards mixed-effect models we used likelihood ratio tests, comparing the full model to a model without a particular variable, to test the significance of each variable’s effect^46^. We tested the significance of the estimated differences between levels within categorical fixed effects using post-hoc Tukey’s tests. We did not reduce our model further due to the issues with stepwise model reduction techniques^46–48^. Correlations between variables fitted in models as fixed effects were lower than the levels previously shown to cause model fitting issues such as variance inflation in effect estimates (*r* < 0.6 in all cases^49^). We performed all analyses in R (R Core Team, 2017^50^), fitting generalized linear mixed-effects models using the lme4 package^51^, Cox proportional hazards models using the coxme package^52^, performing Tukey’s test using the multcomp^53^ and lsmeans packages^54^, and calculating model *r*^2^ values using the MuMIn package^55^.

### Ethics statement

All methods received prior approval from Uganda Wildlife Authority and Uganda National Council for Science and Technology. Ethical oversight and approval for this study was provided by the University of Exeter Ethical Review Committee.

### Reporting summary

Further information on research design is available in the Nature Research Reporting Summary linked to this article.

## Supplementary information

Supplementary Information

Reporting Summary

## Data Availability

The data supporting this study are available from the Figshare repository at 10.6084/m9.figshare.14459151. Source data are provided with this paper.

## Source data

Source Data

## Code availability

The analysis code supporting this study are available from the Figshare repository at 10.6084/m9.figshare.14459151.
