# Supplementary material for: A veil of ignorance can promote fairness in a mammal society
Source: Nat Commun. 2021 Jun 23;12:3717. doi: 10.1038/s41467-021-23910-6 (PMC8222408; doi:10.1038/s41467-021-23910-6)
Supplement: Supplementary file 1 — Supplementary Information [file 41467_2021_23910_MOESM1_ESM.pdf]

A veil of ignorance can promote fairness in a mammal society

Marshall et al

SUPPLEMENTARY INFORMATION

Contents

A. Model Details **2**

No veil 2

Behind the veil 3

Can relatedness between mothers lead to offspring exchange? 4

Supplementary Figures 1-4: model plots 8

B. Supplementary results and tables **12**

Maternal weight change 12

Adult escorting effort 12

Pup weight and growth 13

Escorting received by pups 13

Pup survival 14

Supplementary Figure 5: experimental design schematic 15

Supplementary Tables 1-7: sample size and statistical results 16

Supplementary References 23

A. Model Details

As stated in the main text, we suppose that two unrelated but cooperatively-breeding mothers have each given birth to a single offspring. As a result of differences in pre-natal parental investment, the two young may differ in initial size, denoted x_i for offspring i (we are concerned here with the impact of post-natal care on pre-existing inequalities among young, so we treat the initial sizes of the offspring as given). After birth, each mother must take on the post-natal care of one or other of the two young, leading either to an outcome in which each raises her own offspring, or to an outcome in which each raises the other's offspring. A mother may choose to invest any non-negative level of effort, denoted y_j for mother j, in caring for the offspring that she ends up raising. The survival of offspring i, when raised by mother j, is equal to $b(x_i + y_j)$, a smoothly increasing but decelerating function of the total investment it receives pre- and post-natally, while the mother incurs a cost to her future reproductive success $c(k_j;\, y_j)$ that is a smoothly increasing and accelerating function of y_j. The parameter k_j determines how steeply the cost of care increases with investment for mother j. For the results shown in Figure 1 and discussed in the main text we assume $b(x) = x\,(1 - x/2)$ (for $x \leq 1$) and $c(k;\, y) = k\, y^2$.

No veil

Consider, first, the situation in which the maternity of each offspring is known (i.e. there is no veil of ignorance). If each mother cares for her own offspring, then the payoff to mother i is given by

$$W_i = b(x_i + y_i) - c(k_i; y_i)$$

and the equilibrium level of post-natal investment by mother i, y_i^*, which maximises this payoff, satisfies

$$\left.\frac{\partial W_i}{\partial y_i} = 0\right|_{y_i = y_i^*}$$

For the case considered in Figure 1 and the main text, in which $b(x) = x\,(1 - x/2)$ (where we assume that $x_1, x_2 \leq 1$) and $c(k;\, y) = k\, y^2$, this gives

$$y_i^* = \frac{1 - x_i}{1 + 2k_i}$$

yielding an equilibrium payoff W_i^* of

$$W_i^* = \frac{1 + 2k_i x_i (2 - x_i)}{2 + 4k_i} \qquad \text{(S1)}$$

By contrast, if each mother cares for the other's offspring, then the payoff to mother i is given by

$$W_i = b\left(x_i + y_j\right) - c(k_i; y_i)$$

where y_j denotes the investment in post-natal care by the non-focal mother). The equilibrium level of post-natal investment y_i^* by mother *i*, is then given by

$$y_i^* = 0$$

(since her own offspring does not benefit from her investment), yielding an equilibrium payoff W_i^* of

$$W_i^* = 0 \tag{S2}$$

Comparing (S1) and (S2), we see that both mothers obtain a higher payoff if each cares for her own offspring rather than the other's offspring (since in the latter case, both do best to refrain from investing any effort in care, leading to the death of both young).

Behind the veil

Now consider the situation in which the maternity of the young is concealed behind a veil of ignorance. Under these circumstances, the two offspring can be distinguished only by their initial sizes, and we will write x_{hi} and x_{lo} ($< x_{\text{hi}}$) for the sizes of the larger and of the smaller young, respectively (where we assume that $x_{\text{hi}} \neq x_{\text{lo}}$ and that each offspring is equally likely to be derived from either mother). If mother 1 cares for the larger offspring and mother 2 for the smaller, they obtain payoffs

$$W_1 = \tfrac{1}{2}b(x_{hi} + y_1) + \tfrac{1}{2}b(x_{lo} + y_2) - c(k_1; y_1)$$

$$W_2 = \tfrac{1}{2}b(x_{hi} + y_1) + \tfrac{1}{2}b(x_{lo} + y_2) - c(k_2; y_2)$$

and the equilibrium levels of post-natal investment by the two mothers, y_1^* and y_2^*, satisfy

$$\frac{\partial W_i}{\partial y_i} = 0\Big|_{y_i = y_i^*}$$

For the case considered in Figure 1 and the main text, in which b(x) = $x\,(1 - x/2)$ (where we assume that x_{hi}, $x_{\text{lo}} \leq 1$) and $c(y; k) = k\,y^2$, this gives

$$y_1^* = \frac{1-x_{\text{hi}}}{1+4k_1},\ y_2^* = \frac{1-x_{\text{lo}}}{1+4k_2}$$

yielding payoffs

$$W_1^* = \frac{1+4k_1x_{\text{hi}}(2-x_{\text{hi}})}{4+16k_1} + \frac{\big(1+4k_2(2-x_{\text{lo}})\big)(1+4k_2x_{\text{lo}})}{4(1+4k_2)^2} \tag{S3a}$$

$$W_2^* = \frac{1+4k_2 x_{\mathrm{lo}}(2-x_{\mathrm{lo}})}{4+16k_2} + \frac{\left(1+4k_1(2-x_{\mathrm{hi}})\right)(1+4k_1 x_{\mathrm{hi}})}{4(1+4k_1)^2} \quad \text{(S3b)}$$

An equivalent calculation for the alternative scenario in which mother 1 cares for the smaller offspring and mother 2 for the larger, gives

$$y_1^* = \frac{1-x_{\mathrm{lo}}}{1+4k_1},\ y_2^* = \frac{1-x_{\mathrm{hi}}}{1+4k_2}$$

yielding payoffs

$$W_1^* = \frac{1+4k_1 x_{\mathrm{lo}}(2-x_{\mathrm{lo}})}{4+16k_1} + \frac{\left(1+4k_2(2-x_{\mathrm{hi}})\right)(1+4k_2 x_{\mathrm{hi}})}{4(1+4k_2)^2} \quad \text{(S4a)}$$

$$W_2^* = \frac{1+4k_2 x_{\mathrm{hi}}(2-x_{\mathrm{hi}})}{4+16k_2} + \frac{\left(1+4k_1(2-x_{\mathrm{lo}})\right)(1+4k_1 x_{\mathrm{lo}})}{4(1+4k_1)^2} \quad \text{(S4b)}$$

Comparing (S3) and (S4), we find that if

$$k_1 < \frac{4k_2^2}{1+8k_2} \quad \text{(S5)}$$

then both mothers obtain a greater payoff when mother 1 (who under these circumstances enjoys lower costs of care) cares for the smaller offspring, while if

$$k_2 < \frac{4k_1^2}{1+8k_1} \quad \text{(S6)}$$

then both mothers obtain a greater payoff when mother 2 (who under these circumstances enjoys lower costs of care) cares for the smaller offspring. These are the shaded zones of agreement in Figure 1 in the main text. Note that since each offspring is equally likely to be derived from either mother, the mutually preferred outcome in these zones is as likely to result in each mother caring for the other's offspring as it is to result in each caring for her own. Hence, in the main text, we refer to the outcome as 'care according to need' rather than 'care according to parentage'. If neither (S5) nor (S6) is met, then each mother obtains a greater payoff when the other cares for the smaller offspring; this is the unshaded zone of disagreement in Figure 1 in the main text.

Can relatedness between mothers lead to offspring exchange?

We have seen, above, that a veil of ignorance over parentage can lead (within the zones of agreement) to 'care according to need', with mothers mutually preferring an outcome in which each is as likely to care for the other's offspring as for her own. Here, we consider whether

relatedness between mothers might also lead to a mutually preferable outcome in which each cares for the other's offspring, i.e. to exchange of young, even when parentage is known.

Let *r* denote the coefficient of relatedness between the two mothers. We assume that each behaves (at evolutionary equilibrium) so as to maximise the sum of her own direct fitness payoff from current and future young, plus *r* times the direct fitness payoff to the other mother (a 'simplified' application of Hamilton's rule; see e.g. ref[1] p120).

If each mother cares for her own offspring, then the relevant sum for mother 1 is given by

$$W_1 = b(x_1 + y_1) - c(k_1; y_1) + rb(x_2 + y_2) - rc(k_2; y_2)$$

and for mother 2 by

$$W_2 = b(x_2 + y_2) - c(k_2; y_2) + rb(x_1 + y_1) - rc(k_1; y_1)$$

and the equilibrium levels of post-natal investment y_1^* and y_2^*, which satisfy

$$\frac{\partial w_i}{\partial y_i} = 0\Big|_{y_i = y_i^*} \quad \text{for } i = 1,2,$$

are given by

$$y_i^* = \frac{1 - x_i}{1 + 2k_i},$$

leading to equilibrium payoff sums of

$$W_1^* = \frac{1 + 2k_1 x_1(2 - x_1)}{2 + 4k_1} + r\frac{1 + 2k_2 x_2(2 - x_2)}{2 + 4k_2} \quad \text{(S7a)}$$

$$W_2^* = \frac{1 + 2k_2 x_2(2 - x_2)}{2 + 4k_2} + r\frac{1 + 2k_1 x_1(2 - x_1)}{2 + 4k_1} \quad \text{(S7b)}$$

By contrast, if each mother cares for the other's offspring, then the relevant payoff sum for mother 1 is given by

$$W_1 = b(x_1 + y_2) - c(k_1; y_1) + rb(x_2 + y_1) - rc(k_2; y_2)$$

and for mother 2 by

$$W_2 = b(x_2 + y_1) - c(k_2; y_2) + rb(x_1 + y_2) - rc(k_1; y_1),$$

and the equilibrium levels of post-natal investment y_1^* and y_2^*, which satisfy

$$\frac{\partial w_i}{\partial y_i} = 0\Big|_{y_i = y_i^*} \quad \text{for } i = 1,2,$$

are given by

$$y_1^* = \frac{r(1-x_2)}{1+2k_1},\ y_2^* = \frac{r(1-x_1)}{1+2k_2},$$

leading to equilibrium payoff sums of

$$W_1^* = \frac{r(r+4k_2)+4k_2^2 s_1(2-s_1)}{2(r+2k_2)^2} - \frac{rk_1(1-s_2)^2}{(r+2k_1)^2} + r\left(\frac{r(r+4k_1)+4k_1^2 s_1(2-s_1)}{2(r+2k_1)^2} - \frac{rk_2(1-s_1)^2}{(r+2k_2)^2}\right) \quad \text{(S8a)}$$

$$W_2^* = \frac{r(r+4k_1)+4k_1^2 s_1(2-s_1)}{2(r+2k_1)^2} - \frac{rk_1(1-s_2)^2}{(r+2k_1)^2} + r\left(\frac{r(r+4k_2)+4k_2^2 s_1(2-s_1)}{2(r+2k_2)^2} - \frac{rk_1(1-s_2)^2}{(r+2k_1)^2}\right) \quad \text{(S8b)}$$

Comparing (S7) and (S8) we can then determine whether, and under what circumstances, both parents favour exchange of young (assuming that exchange will occur only if it is in the favour of both).

Whereas the outcome of the model behind the veil depended only on the costs of care to each mother, i.e. on the parameters k_1 and k_2, in this case, the outcome depends also on the relatedness between mothers *r,* and on the initial sizes of the offspring, x_1 and x_2. In general, a mutual preference for exchange of young is more likely when the costs of care differ markedly between the two mothers, when relatedness is high, and when the two offspring differ markedly in size. In each of the Supplementary Figures 1 to 4 below, we show the combinations of k_1 and k_2 for which both mothers favour exchange (as in Figure 1 in the main text), for $r = 0.125$, 0.25, 0.5 and 0.75 (note that, as shown above, when $r = 0$ and the mothers are unrelated, each always prefers to care for her own offspring regardless of other parameter values). The four figures show results for increasing degrees of initial size asymmetry between the offspring (assuming in all cases that $x_2 > x_1$, i.e. that the larger offspring is born to parent 2; results when $x_1 > x_2$ are simply a mirror image of those shown). Supplementary Figure 1 assumes the smallest degree of asymmetry, equivalent to that assumed at point A in Figure 1 in the main text ($x_1 = 0.15$, $x_2 = 0.25$). Supplementary Figure 2 assumes a three-fold asymmetry equivalent to that assumed at point B in Figure 1 in the main text ($x_1 = 0.1$, $x_2 = 0.3$), which is already quite large compared to the asymmetries observed in our experimental study. In Supplementary Figure 3 the asymmetry is still larger, at five-fold ($x_1 = 0.1$, $x_2 = 0.5$); and in Supplementary Figure 4 seven-fold ($x_1 = 0.1$, $x_2 = 0.7$).

Our results suggest that exchange of young is only likely for higher levels of relatedness and greater size asymmetries than are plausible for the mongoose study system, where relatedness between mothers is typically 0.24 (median, IQR = 0.05-0.37, n = 1,134 dyads across 49 breeding attempts) and size asymmetries typically 1.44 ± 0.29 (mean ± s.d. largest/smallest pup weight under 50 days old, n = 52 litters). For instance, even assuming a three-fold difference in initial size between offspring, exchange is only feasible for relatedness values of ~0.7 and above (and

then is only likely if parents differ markedly in the costs of care). For exchange of young to prove mutually beneficial given a more plausible relatedness of 0.25 requires around a seven-fold difference in initial size between young (and again a marked difference between parents in the costs of care).

Supplementary Figure 1

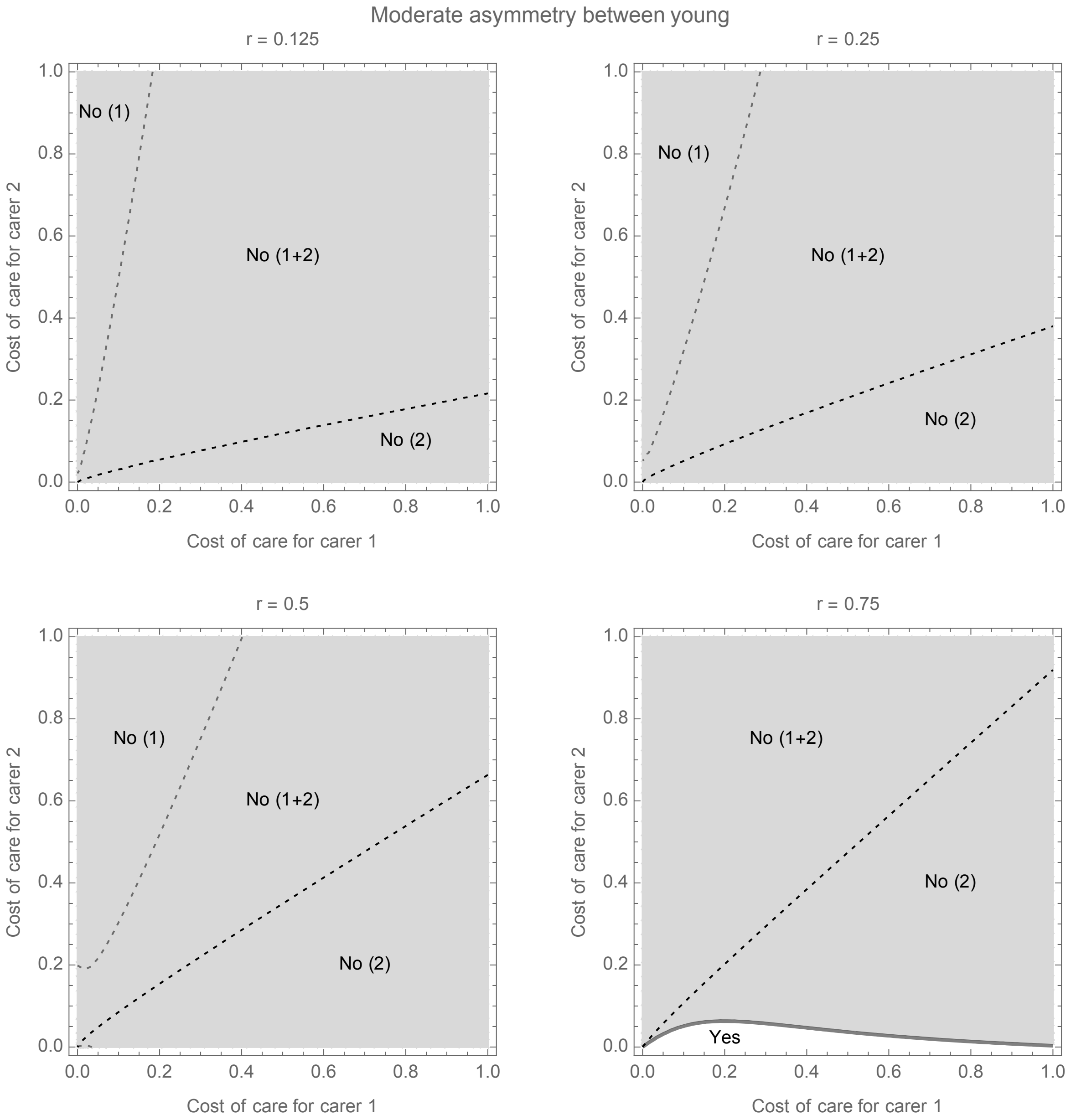

Supplementary Figure 1. Combinations of k_1 and k_2 (costs of care for mothers 1 and 2) for which both mothers favour exchange (as in figure 1 in the main text), for r = 0.125, 0.25, 0.5 and 0.75, assuming initial offspring sizes of x_1 = 0.15 and x_2 = 0.25 (as at point A of Figure 1 in the main text). Note that, as shown above, when r = 0 and the mothers are unrelated, each always prefers to care for her own offspring regardless of other parameter values. The unshaded region (labelled 'yes') in each panel corresponds to the zone within which both mothers stand to gain by exchanging young; in the shaded region (labelled 'no'), one or both of the mothers stands to gain if each cares for its own offspring (the numbers in parentheses following the 'no' indicating for which parent(s) this is the case).

Supplementary Figure 2

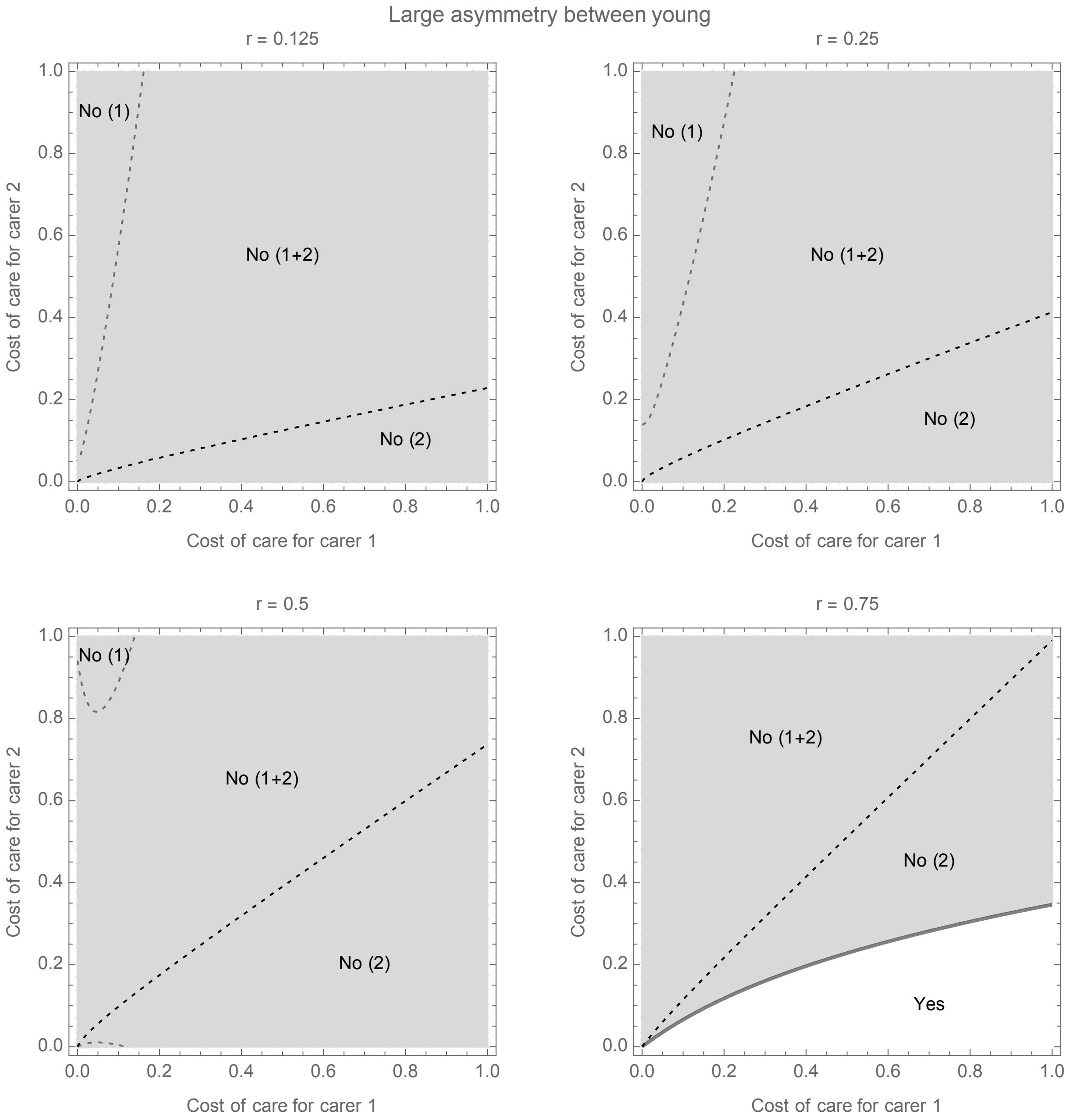

Supplementary Figure 2. Combinations of k_1 and k_2 (costs of care for mothers 1 and 2) for which both mothers favour exchange (as in Figure 1 in the main text), for r = 0.125, 0.25, 0.5 and 0.75, assuming initial offspring sizes of x_1 = 0.1 and x_2 = 0.3 (as at point B of Figure 1 in the main text). Note that, as shown above, when r = 0 and the mothers are unrelated, each always prefers to care for her own offspring regardless of other parameter values. The unshaded region (labelled 'yes') in each panel corresponds to the zone within which both mothers stand to gain by exchanging young; in the shaded region (labelled 'no'), one or both of the mothers stands to gain if each cares for its own offspring (the numbers in parentheses following the 'no' indicating for which parent(s) this is the case).

Supplementary Figure 3

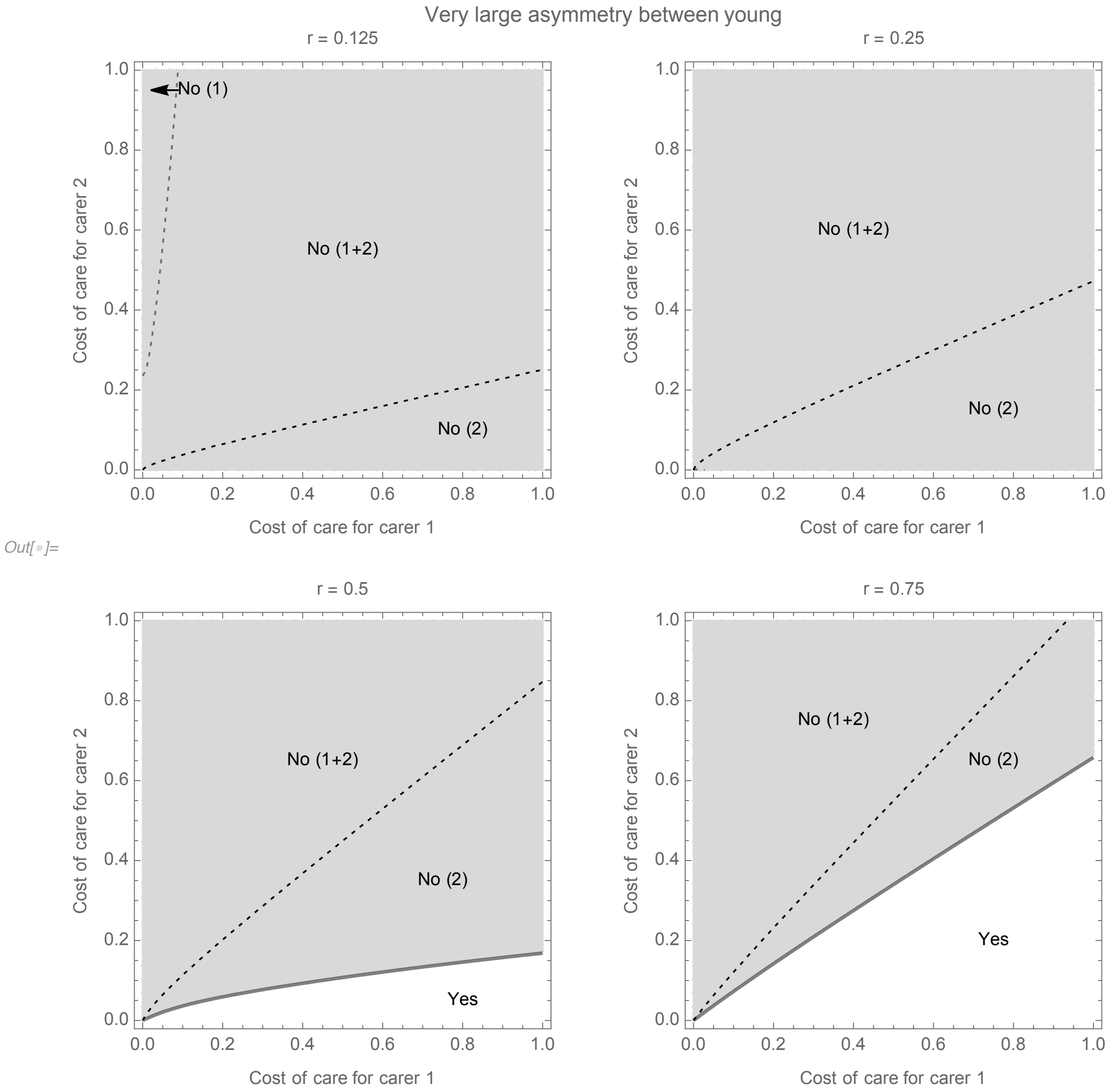

Supplementary Figure 3. Combinations of k_1 and k_2 (costs of care for mothers 1 and 2) for which both mothers favour exchange (as in Figure 1 in the main text), for r = 0.125, 0.25, 0.5 and 0.75, assuming initial offspring sizes of x_1 = 0.1 and x_2 = 0.5. (note that, as shown above, when r = 0 and the mothers are unrelated, each always prefers to care for her own offspring regardless of other parameter values). The unshaded region (labelled ‘yes’) in each panel corresponds to the zone within which both mothers stand to gain by exchanging young; in the shaded region (labelled ‘no’), one or both of the mothers stands to gain if each cares for its own offspring (the numbers in parentheses following the ‘no’ indicating for which parent(s) this is the case).

Supplementary Figure 4

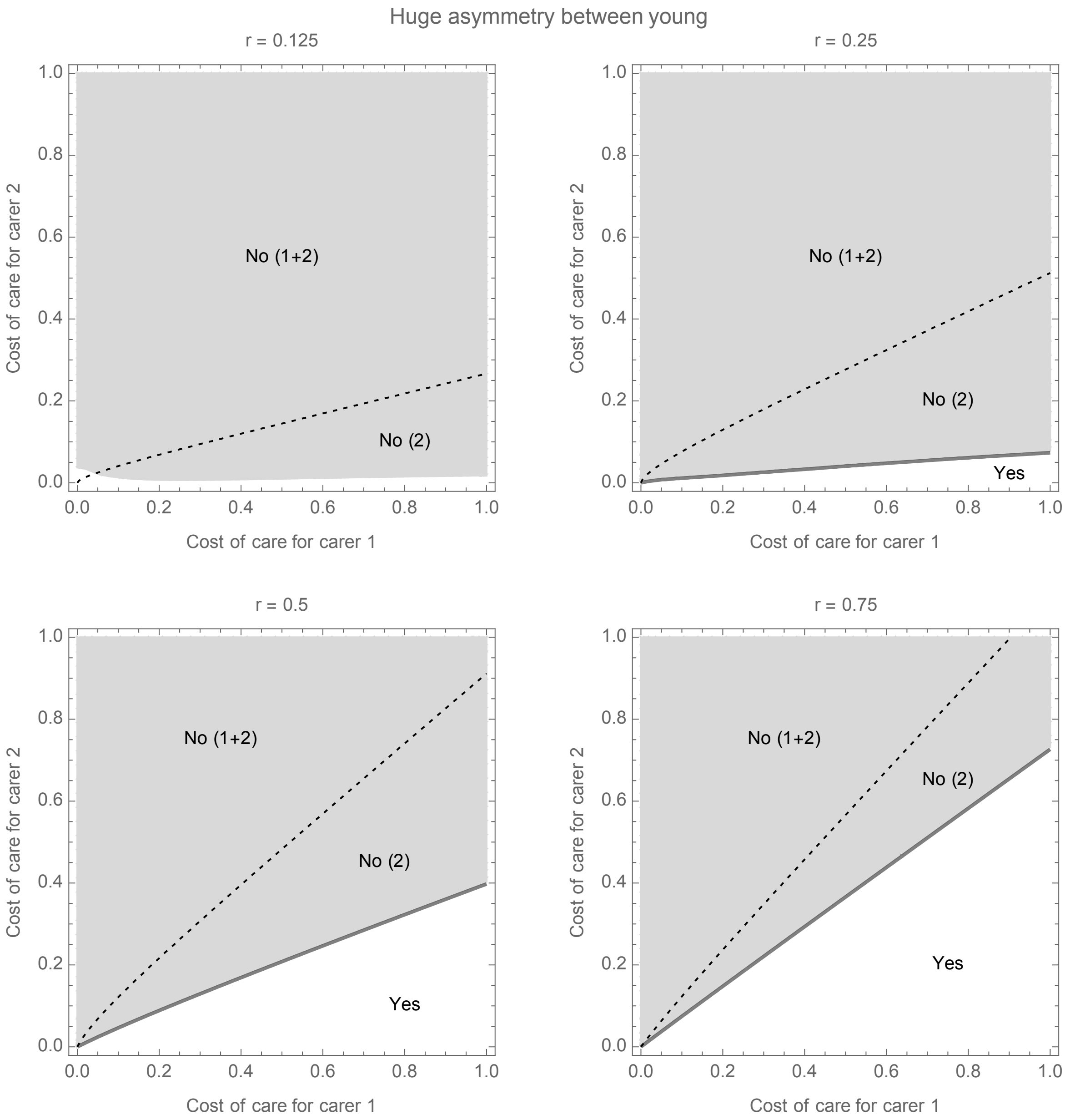

Supplementary Figure 4. Combinations of k_1 and k_2 (costs of care for mothers 1 and 2) for which both mothers favour exchange (as in Figure 1 in the main text), for r = 0.125, 0.25, 0.5 and 0.75, assuming initial offspring sizes of x_1 = 0.1 and x_2 = 0.7. (note that, as shown above, when r = 0 and the mothers are unrelated, each always prefers to care for her own offspring regardless of other parameter values). The unshaded region (labelled 'yes') in each panel corresponds to the zone within which both mothers stand to gain by exchanging young; in the shaded region (labelled 'no'), one or both of the mothers stands to gain if each cares for its own offspring (the numbers in parentheses following the 'no' indicating for which parent(s) this is the case).

B. Supplementary results and tables

Maternal weight change

Experimental category (fed, non-fed) predicted variation in maternal weight change from pre-pregnancy baseline during pregnancy and post-pregnancy periods, but not during the escorting period (Supplementary Table 2). During pregnancy, fed females proportionally gained more weight than non-fed females and females in unmanipulated breeding attempts (post-hoc Tukey's test, PHT. fed vs non-fed: $z = 2.42$, $p = 0.039$; fed vs unmanipulated: $z = 2.71$, $p = 0.017$), but non-fed and unmanipulated females gained similar amounts of weight (PHT. $z = 1.19$, $p = 0.45$). Fed females remained heavier than non-fed females in the post-pregnancy period (PHT. $z = 2.68$, $p = 0.019$), but there was no significant difference between fed and unmanipulated females at this stage, nor between non-fed and unmanipulated females compared to their pre-pregnancy baselines (PHT. fed vs unmanipulated: $z = 1.90$, $p = 0.13$; non-fed vs unmanipulated: $z = 0.51$, $p = 0.86$). By the escorting period fed, non-fed and unmanipulated females all had similar weight changes compared to pre-pregnancy (PHT. fed vs non-fed: $z = 1.11$, $p = 0.51$; fed vs unmanipulated: $z = 0.03$, $p = 1.00$; non-fed vs unmanipulated: $z = 0.96$, $p = 0.60$).

Adult escorting effort

Experimental category (females) and breeding attempt type (males) explained a significant proportion of the variation in individuals' total escorting effort within each breeding attempt (Supplementary Table 3). Fed females escorted pups more than non-fed and unmanipulated females (PHT. fed vs non-fed: $z = 3.39$, $p = 0.002$; fed vs unmanipulated: $z = 5.11$, $p = 6.33 \times 10^{-7}$), but the non-fed females and females in unmanipulated breeding attempts did not differ in their escorting effort (PHT, $z = 1.43$, $p = 0.32$). Males escorted pups more in experimentally manipulated breeding attempts than in unmanipulated breeding attempts (PHT. $z = 2.76$, $p = 0.006$). Both males and females escorted pups less when there were more adults per pup in the group, but age did not affect escorting effort in either case (Supplementary Table 3).

Female escorting allocation depended on both their and the pup's experimental category (Supplementary Table 4). Fed females invested more escorting effort in control pups than

treatment pups (PHT, z = 2.33, p = 0.02), but the amount of escorting effort provided by non-fed females did not differ between control and treatment pups (PHT, z = 0.16, p = 0.88). Males' escorting effort did not differ between treatment and control pups (PHT, z = 1.65, p = 0.10; Supplementary Table 4). Relatedness between escorts and pups was lower in experimentally manipulated litters compared to unmanipulated litters (experimental: median = 0.11, IQR = -0.04-0.30, n = 100 dyads across 10 litters; unmanipulated: median = 0.22, IQR = 0.06-0.36, n = 109 dyads across 11 litters; Mann-Whitney U = 6,599, p = 0.009). There was no difference in the relatedness of non-escort adults and pups in experimental and unmanipulated litters (experimental: median = 0.18, IQR = 0.003-0.32, n = 843 dyads across 10 litters; unmanipulated: median = 0.20, IQR = 0.04-0.34, n = 865 dyads across 11 litters; Mann-Whitney U = 381647, p = 0.09)

Pup weight and growth

Our growth models estimated differences in birth weight between treatment and control pups (mean ± s.d. predicted birth weights: treatment = 164.9 ± 3.5g, control = 142.0 ± 3.2g; β ± s.e. = 0.15 ± 0.071, z = 2.13, p = 0.033, Supplementary Table 5), but not between pups from unmanipulated litters (143.5 ± 6.5g) and treatment or control pups (treatment vs unmanipulated: z = 1.26, p = 0.21; control vs unmanipulated: z = 0.41, p = 0.68). Pup growth across the escorting period also depended on experimental category (age x experimental category in Supplementary Table 5). Control pups grew faster than treatment pups (PHT. β ± s.e. = 0.0022 ± 0.0008, z = 2.68, p = 0.020), but growth rates did not differ between control and unmanipulated pups (PHT. β ± s.e. = 0.0003 ± 0.0010, z = 0.34, p = 0.94) and treatment and unmanipulated pups (PHT. β ± s.e. = 0.0019 ± 0.0011, z = 1.81, p = 0.17). Pup weights in manipulated litters were more variable than in unmanipulated litters at the start of the escorting period but there was no difference by the end of escorting (relative within-litter variance in manipulated versus unmanipulated litters in: pups aged 30-60 days, U = 16, p = 0.029; pups aged 60-90 days, U = 13, p = 0.38).

Escorting received by pups

Pup experimental category predicted the total amount of escorting that a pup received across the escorting period as well as the rate they were fed by their escort (Supplementary Table 6). Control pups received more escorting than treatment pups and pups born in unmanipulated litters (PHT. control vs treatment: $\beta \pm$ s.e. = 0.81 ± 0.23, z = 3.59, p = 9.13×10^{-4}; control vs unmanipulated: $\beta \pm$ s.e. = 1.34 ± 0.42, z = 3.19, p = 0.0038), but the amount of escorting received by treatment and unmanipulated pups did not differ (PHT. $\beta \pm$ s.e. = 0.53 ± 0.41, z = 1.28, p = 0.39). Control pups were also fed more often by their escorts than treatment pups (PHT. $\beta \pm$ s.e. = 0.85 ± 0.28, z = 3.09, p = 0.005). Pups born in unmanipulated litters were fed more often by their escorts than treatment pups (PHT. $\beta \pm$ s.e. = 0.58 ± 0.23, z = 2.49, p = 0.032), but were fed at the same rate as control pups (PHT. $\beta \pm$ s.e. = 0.27 ± 0.19, z = 1.42, p = 0.33).

Pup survival

Pups from the different experimental categories did not differ significantly in their survival to 90, 180 or 365 days old (Supplementary Table 7).

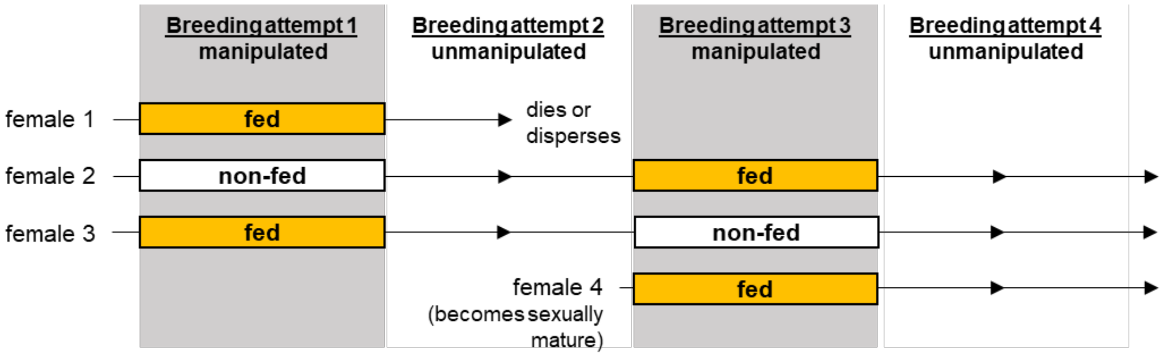

Supplementary Figure 5. An example schematic of the experimental design within each banded mongoose group. Breeding attempt 1: three females are pregnant and females 1 and 3 are assigned as 'fed' females, whilst female 2 is left as the 'non-fed' control. Breeding attempt 2: unmanipulated breeding attempt with no females fed to allow the effects of the provisioning in breeding attempt 1 to dissipate. During this period female 1 leaves the group (through death or dispersal) and female 4 becomes sexually mature. Breeding attempt 3: Females 2 and 3 are paired and assigned the opposite groups to the groups they were assigned in breeding attempt 1. Female 4 is unpaired and is randomly assigned to the fed category (though she could just as easily have been assigned to the non-fed category). Breeding attempt 4: unmanipulated breeding attempt with no females fed to allow the effects of the provisioning in breeding attempt 3 to dissipate.

Supplementary Table 1. Sample sizes used in the statistical models.

	Number of:			
Response variable	Records	Individuals	Breeding attempts	Social groups
Female weight change from conception (67-74 days before birth) to:				
- pregnancy (mean ± s.d. = 10.8 ± 4.25 days before birth)	45	23	17	4
- post-pregnancy (mean ± s.d. = 15.6 ± 2.22 days after birth)	41	21	15	4
- escorting (mean ± s.d. = 77.6 ± 14.9 days after birth)	43	22	17	4
Total escorting effort in each breeding attempt by:				
- adult females	34	23	10	3
- adult males	110	44	19	3
Escorting allocation among pups by:				
- adult females	21	14	5	4
- adult males	56	35	9	4
Pup weight during escorting period	293	68	18	3
Total escorting received by each pup		71	12	3
Rate of pup feeding by escorts	79	43	11	3
Pup survival to 90, 180 and 365 days		128	23	4

Supplementary Table 2. Linear mixed effects models predicting the effect of prenatal provisioning on maternal percentage weight change since conception. β and standard error (s.e.) denote the parameter estimates, and uncertainty, for each variable. χ^2 and p values are from likelihood ratio tests and r^2 values are the conditional values for each model[2].

Percentage change in weight from pre-pregnancy baseline to:	Explanatory variable	β	s.e.	χ^2	p	r^2
Pregnancy						0.77
	intercept	1.09	0.04			
	experimental category[a]			10.08	0.0065	
	non-fed	0.07	0.06			
	fed	0.16	0.06			
Post-pregnancy						0.91
	intercept	0.98	0.04			
	experimental category[a]			7.06	0.029	
	non-fed	0.03	0.06			
	fed	0.11	0.06			
Escorting						0.43
	intercept	1.04	0.04			
	experimental category[a]			1.49	0.47	
	non-fed	-0.06	0.06			
	fed	0.001	0.05			

[a]reference category = females in unmanipulated breeding attempts

Supplementary Table 3. Binomial mixed effects models predicting the effect of prenatal provisioning on female and male escorting effort per breeding attempt. β and standard error (s.e.) denote the parameter estimates, and uncertainty, for each variable. χ^2 and p values are from likelihood ratio tests and r^2 values are the conditional values for each model[2].

	Explanatory variable	β	s.e.	χ^2	p	r^2
Females						0.72
	intercept	1.55	1.77			
	experimental category[a]			16.48	0.0003	
	non-fed	0.47	0.33			
	fed	1.91	0.37			
	adult:pup ratio	-0.74	0.21	5.69	0.017	
	female age	0.12	0.13	0.73	0.39	
Males						0.36
	intercept	0.05	0.47			
	breeding attempt (manipulated)[a]	0.97	0.35	5.71	0.02	
	adult:pup ratio	-0.26	0.12	3.98	0.05	
	male age	0.005	0.08	0.004	0.95	

[a]reference category = unmanipulated litters

Supplementary Table 4. Binomial mixed effects models predicting the effect of prenatal provisioning on female and male escorting allocation between control and treatment pups. β and standard error (s.e.) denote the parameter estimates, and uncertainty, for each variable. χ^2 and p values are from likelihood ratio tests and r^2 values are the conditional values for each model[2].

	Explanatory variable	β	s.e.	χ^2	p	r^2
Females						0.48
	intercept	-1.92	1.05			
	female experimental category (fed)[a]	2.98	0.89			
	pup experimental category (treatment)[b]	-0.12	0.78			
	female experimental category (fed)[a] x pup experimental category (treatment)[b]	-2.40	1.19	4.22	0.04	
	adult:pup ratio	-0.14	0.12	1.33	0.25	
	female age	0.43	0.46	0.85	0.36	
	pup sex (male)[c]	-0.34	0.88	0.14	0.71	
Males						0.70
	intercept	1.78	1.30			
	pup experimental category (treatment)[b]	1.31	0.79	2.72	0.10	
	adult:pup ratio	-0.06	0.13	0.18	0.67	
	male age	-0.38	0.15	5.59	0.02	
	pup sex (male)[c]	-0.40	0.74	0.29	0.59	

reference categories = [a]non-fed females, [b]control pups, [c]female

Supplementary Table 5. Poisson log-normal mixed effects model predicting the effect of prenatal provisioning on pup weight and growth during the escorting period (age 30-90 days). β and standard error (s.e.) denote the parameter estimates, and uncertainty, for each variable. χ^2 and p values are from likelihood ratio tests and r^2 values are the conditional values for the model[2].

Explanatory variable	β	s.e.	χ^2	p	r^2
					0.96
intercept	4.99	0.075			
pup age	0.014	5.57×10^{-4}			
pup experimental category[a]					
treatment	0.15	0.071			
unmanipulated	0.036	0.086			
pup age x pup experimental category[a]			7.52	0.023	
treatment	-0.002	8.46×10^{-4}			
unmanipulated	3.44×10^{-4}	0.001			
adult:pup ratio	-0.014	0.005	8.40	0.004	
pup sex (male)[b]	-0.029	0.033	0.79	0.38	

reference categories = [a]control pups, [b]female

Supplementary Table 6. Mixed effects model predicting the effect of prenatal provisioning on the total escorting received by pup (binomial model) and the rate they were fed by escorts (Poisson log-normal model). β and standard error (s.e.) denote the parameter estimates, and uncertainty, for each variable. χ^2 and p values are from likelihood ratio tests and r^2 values are the conditional values for each model[2]

	Explanatory variable	β	s.e.	χ^2	p	r^2
Total escorting received by pups						0.45
	intercept	1.61	1.01			
	pup experimental category[a]			16.98	2.05×10^{-4}	
	treatment	-0.81	0.23			
	unmanipulated	-1.34	0.42			
	adult:pup ratio	0.08	0.19	0.19	0.66	
	pup sex (male)[b]	0.02	0.17	0.02	0.89	
Pup feeding rate (per hour)						0.72
	intercept	2.00	0.40			
	pup experimental category[a]			7.30	0.026	
	treatment	-0.85	0.28			
	unmanipulated	-0.27	0.19			
	pup-escort relationship strength	0.20	0.41	0.24	0.63	

reference category = [a]control pups

Supplementary Table 7. Cox proportional hazards mixed-effects models predicting the effect of prenatal provisioning on pup survival to 90, 180 and 365 days old. β and standard error (s.e.) denote the parameter estimates, and uncertainty, for each variable. χ^2 and p values are from likelihood ratio tests and r^2 values are the likelihood-ratio pseudo values for each model[3].

	Explanatory variable	β	s.e.	χ^2	p	r^2
Survival to 90 days						0.45
	pup experimental category[a]			1.07	0.59	
	treatment	0.43	0.40			
	unmanipulated	0.07	0.86			
	adult:pup ratio	0.17	0.36	0.03	0.86	
	pup sex (male)[b]	-0.11	0.30	0.13	0.72	
Survival to 180 days						0.49
	pup experimental category[a]			2.21	0.33	
	treatment	0.53	0.35			
	unmanipulated	0.08	0.81			
	adult:pup ratio	0.16	0.34	0.06	0.81	
	pup sex (male)[b]	-0.08	0.26	0.09	0.76	
Survival to 365 days						0.47
	pup experimental category[a]			2.51	0.29	
	treatment	0.51	0.34			
	unmanipulated	-0.20	0.70			
	adult:pup ratio	0.15	0.30	0.08	0.78	
	pup sex (male)[b]	0.05	0.25	0.06	0.81	

reference category = [a]control pups

Supplementary references

1 Okasha, S. *Agents and goals in evolution*. (Oxford University Press, 2018).
2 Nakagawa, S., Johnson, P. C. & Schielzeth, H. The coefficient of determination R 2 and intra-class correlation coefficient from generalized linear mixed-effects models revisited and expanded. *Journal of the Royal Society Interface* **14**, 20170213 (2017).
3 Magee, L. R 2 measures based on Wald and likelihood ratio joint significance tests. *The American Statistician* **44**, 250-253 (1990).
