# Supplementary material for: A veil of ignorance can promote fairness in a mammal society
Source: Nat Commun. 2021 Jun 23;12:3717. doi: 10.1038/s41467-021-23910-6 (PMC8222408; doi:10.1038/s41467-021-23910-6)
Supplement: Supplementary file 2 — Reporting Summary [file 41467_2021_23910_MOESM2_ESM.pdf]

nature research

Corresponding author(s): Harry H Marshall, Michael A Cant

Last updated by author(s): Apr 28, 2021

Reporting Summary

Nature Research wishes to improve the reproducibility of the work that we publish. This form provides structure for consistency and transparency in reporting. For further information on Nature Research policies, see our Editorial Policies and the Editorial Policy Checklist.

Statistics

For all statistical analyses, confirm that the following items are present in the figure legend, table legend, main text, or Methods section.

n/a	Confirmed	
☐	☒	The exact sample size (n) for each experimental group/condition, given as a discrete number and unit of measurement
☐	☒	A statement on whether measurements were taken from distinct samples or whether the same sample was measured repeatedly
☐	☒	The statistical test(s) used AND whether they are one- or two-sided<br>*Only common tests should be described solely by name; describe more complex techniques in the Methods section.*
☐	☒	A description of all covariates tested
☐	☒	A description of any assumptions or corrections, such as tests of normality and adjustment for multiple comparisons
☐	☒	A full description of the statistical parameters including central tendency (e.g. means) or other basic estimates (e.g. regression coefficient) AND variation (e.g. standard deviation) or associated estimates of uncertainty (e.g. confidence intervals)
☐	☒	For null hypothesis testing, the test statistic (e.g. F, t, r) with confidence intervals, effect sizes, degrees of freedom and P value noted<br>*Give P values as exact values whenever suitable.*
☒	☐	For Bayesian analysis, information on the choice of priors and Markov chain Monte Carlo settings
☒	☐	For hierarchical and complex designs, identification of the appropriate level for tests and full reporting of outcomes
☐	☒	Estimates of effect sizes (e.g. Cohen's d, Pearson's r), indicating how they were calculated

Our web collection on statistics for biologists contains articles on many of the points above.

Software and code

Policy information about availability of computer code

Data collection	Behavioural and life-history data were collected in the field using Samsung Galaxy tablet computers using the Mongoose 2000 data collection app. This app is freely available through the Google Play Store: https://play.google.com/store/apps/details?id=foam.mongoose&hl=en_SG
Data analysis	The data were analysed using the R statistical platform (version 3.4.1) using the packages lme4 (v1.1-25), coxme (v2.2-14), multcomp (v1.4-10), lsmeans (v2.30-0) and MuMIN (v1.43.17). The analysis code is available from the Figshare repository at dx.doi.org/10.6084/m9.figshare.14459151

For manuscripts utilizing custom algorithms or software that are central to the research but not yet described in published literature, software must be made available to editors and reviewers. We strongly encourage code deposition in a community repository (e.g. GitHub). See the Nature Research guidelines for submitting code & software for further information.

Data

Policy information about availability of data

All manuscripts must include a data availability statement. This statement should provide the following information, where applicable:

- Accession codes, unique identifiers, or web links for publicly available datasets
- A list of figures that have associated raw data
- A description of any restrictions on data availability

The data supporting this study are available from the Figshare repository at dx.doi.org/10.6084/m9.figshare.14459151

Field-specific reporting

Please select the one below that is the best fit for your research. If you are not sure, read the appropriate sections before making your selection.

☐ Life sciences ☐ Behavioural & social sciences ☒ Ecological, evolutionary & environmental sciences

For a reference copy of the document with all sections, see nature.com/documents/nr-reporting-summary-flat.pdf

Ecological, evolutionary & environmental sciences study design

All studies must disclose on these points even when the disclosure is negative.

Study description	We developed an evolutionary model of parental care allocation exploring how ignorance about the relatedness between carers and offspring influenced how carers allocated their care to offspring of different sizes (qualities). We then tested the predictions of this model using a field experiment conducted on a wild banded mongooses (Mungos mungo). This experiment induced early-life asymmetries in mongoose pups by manipulating the resources available to females during pregnancy using targeting provisioning. This involved 101 fed pregnancies (treatment) and 97 non-fed pregnancies (controls) across 34 breeding attempts and 7 mongoose groups. These pregnancies produced 50 pups from fed mothers and 50 pups from non-fed mothers. To test our model's predictions we compared the growth, care received and survival of thee two groups of pups.
Research sample	Our study included data collected from 7 groups of banded mongooses in Queen Elizabeth National Park, Uganda. Our field experiment involved provisioning 101 female pregnancies with a further 97 pregnancies non-provisioned as matched-pair controls (aged 0.81-7.88 years old). These pregnancies produced 100 pups (50 from fed mothers and 50 from non-fed mothers) which emerged from the underground den at around 30 days old (the earliest they are visible for data collection) and from which we collected data growth and behavioural data until 90 days old and survival data until 1 year old.<br><br>As this study was performed on a natural population females were included in our experiment when the study groups naturally reproduced. See Methods and Figure S5 for details of female assignment to experimental groups. This individuals in this sample are representative of the study population.
Sampling strategy	Our study included all groups in our wild study population which were habituated enough to allow targeted provisioning and the collection of detailed behavioural and weight data in the field. Our study was conducted on wild animals which are subject to natural variation in processes such as births, deaths and dispersal. As a result our sample size was determined by these natural processes (and are beyond our control) and so no sample size calculation was performed. We designed our experiment to provide an expected samples size that would be adequate to detect effects based on previous experience of manipulating wild populations at this and other study sites.
Data collection	Data were collected at the Banded Mongoose Research Project on the Mweya Peninsula, Queen Elizabeth National Park, Uganda (0° 12'S, 29°54'E) by Francis Mwanguhya, Solomon Kyabulima, Kenneth Mwesige, Robert Businge and Solomon Ahabyona. This involved visiting the study groups every 1-3 days for at least 20 minutes to collect behavioural, weight and life history data. One to two individuals in each group are fitted with radio collars allowing them to be located. Each group is trapped around every 3 months to monitor reproduction and collect morphometric data and tissue samples.
Timing and spatial scale	The data presented in this paper were collected continuously from August 2013 to September 2016. The data were collected from banded mongoose groups living on the Mweya Peninsula (~ 5km^2) in Queen Elizabeth National Park, Uganda.
Data exclusions	All complete records were included in each analysis. The variable sample size for each analysis (see Table S1) reflects records not being included in an analysis because they lacked data for some variables.
Reproducibility	Our experiment was conducted in 34 separate breeding attempts in seven different mongoose groups. Reproducibility was ensured by the field experiment and data collection being conducted the same field team across the entire study (Francis Mwanguhya, Solomon Kyabulima, Kenneth Mwesige, Robert Businge and Solomon Ahabyona). At the start of this study this field team had a combined 39 years of experience collecting data on mongoose behaviour, weights and life histories, live-trapping mongooses safely and conducting field experiments. They used data collection methods that had been well-established at our field site before the start of this study.
Randomization	Pregnant females were randomly assigned to the fed to non-fed groups within age-matched pairs. See Methods in Supplementary Information for full description (e.g. Figure S5).
Blinding	Our experiment was naturally blinded as pup parentage (and so whether their mothers were experimentally provisioned or not) is only known after DNA analysis which occured separately to, and after, the weight, behavioural and life history data were collected from the pups in the field.

Did the study involve field work? ☒ Yes ☐ No

Field work, collection and transport

Field conditions	Fieldwork was conducted at an equatorial study site in Uganda (see details below). The temperature at this site is relatively stable all year round (27 to 31C). Rainfall varies between around 35mm per month in the dry seasons (Jan-Feb, Jun-Jul) and around 9mmm per month in the wet seasons (Mar-May, Aug-Dec).

Location	Data were collected at the Banded Mongoose Research Project on the Mweya Peninsula, Queen Elizabeth National Park, Uganda (0° 12'S, 29°54'E).
Access & import/export	Research and sample export permissions were granted by the Uganda Wildlife Authority and Uganda National Council for Science and Technology. Permissions to import samples into the UK (for DNA analysis) were granted by the Department foe Environment Food and Rural Affairs (authorisation no. TARP/2015/048).
Disturbance	Our field experiment, field data collection and trapping represent a mild disturbance to our banded mongoose study population. This disturbance was minimised through the use of a highly experienced field team using well-established data collection methods (see 'Reproducibility' above). Its effects were monitored through regular observation and weighing of the study animals. No adverse effects were detected.

Reporting for specific materials, systems and methods

We require information from authors about some types of materials, experimental systems and methods used in many studies. Here, indicate whether each material, system or method listed is relevant to your study. If you are not sure if a list item applies to your research, read the appropriate section before selecting a response.

Materials & experimental systems

n/a	Involved in the study
☒	☐ Antibodies
☒	☐ Eukaryotic cell lines
☒	☐ Palaeontology and archaeology
☐	☒ Animals and other organisms
☒	☐ Human research participants
☒	☐ Clinical data
☒	☐ Dual use research of concern

Methods

n/a	Involved in the study
☒	☐ ChIP-seq
☒	☐ Flow cytometry
☒	☐ MRI-based neuroimaging

Animals and other organisms

Policy information about studies involving animals; ARRIVE guidelines recommended for reporting animal research

Laboratory animals	The study did not involve laboratory animals
Wild animals	The study involved the observation and live-trapping of wild banded mongooses (Mungos mungo). The study groups include a mix of male and females with individuals observed ranging from around 30 days old up to many years old (max. 11 years). As these are wild animals the age and sex composition of the study groups varied across the study period due to natural mortality (e.g. predation, disease), birth and dispersal processes.<br><br>Live-trapping involved groups being trapped using box traps (67 x 23 x 23 cm; Tomahawk Live Trap Co., Tomahawk, WI, USA), one trap per individual, and anaesthetised using isoflurane applied through a silicon face mask. When they were first trapped (either as newly emerged pups or newly arrived immigrants) a 2mm skin sample was taken from individuals' tail tips using sterilised scissors. The small wound was then treated with antiseptic. After trapping each animal was placed back in its trap, provided with water ad libitum, covered with black cloth and allowed to recover. Once fully mobile all animals from the group were released together at the point they were trapped and monitored for 20 minutes. This study did not involve any animals being killed.
Field-collected samples	Tissue samples for DNA analysis were collected in ethanol-filled eppendorf tubes. In the field these tubes were stored in opaque boxes in a -20 freezer to minimise ethanol evaporation. These samples were transported to the UK by members of the research team on commercial flights. During transport sample boxes were stored in insulated cool bags with ice blocks. Once in the UK sample boxes were stored in a -20 freezer until being analysed in the laboratory. This laboratory analysis destroyed the samples.
Ethics oversight	All methods received prior approval from Uganda Wildlife Authority and Uganda National Council for Science and Technology. Ethics oversight and approval was provided by the University of Exeter Ethical Review Committee.

Note that full information on the approval of the study protocol must also be provided in the manuscript.
